# MicroRNAs in the Onset of Schizophrenia

**DOI:** 10.3390/cells10102679

**Published:** 2021-10-06

**Authors:** Kristen T. Thomas, Stanislav S. Zakharenko

**Affiliations:** Department of Developmental Neurobiology, St. Jude Children’s Research Hospital, Memphis, TN 38105, USA

**Keywords:** schizophrenia, microRNA, adolescence, neurodevelopment, age of onset, miR-29, miR-132-3p, miR-137, 22q11.2 deletion syndrome, DGCR8

## Abstract

Mounting evidence implicates microRNAs (miRNAs) in the pathology of schizophrenia. These small noncoding RNAs bind to mRNAs containing complementary sequences and promote their degradation and/or inhibit protein synthesis. A single miRNA may have hundreds of targets, and miRNA targets are overrepresented among schizophrenia-risk genes. Although schizophrenia is a neurodevelopmental disorder, symptoms usually do not appear until adolescence, and most patients do not receive a schizophrenia diagnosis until late adolescence or early adulthood. However, few studies have examined miRNAs during this critical period. First, we examine evidence that the miRNA pathway is dynamic throughout adolescence and adulthood and that miRNAs regulate processes critical to late neurodevelopment that are aberrant in patients with schizophrenia. Next, we examine evidence implicating miRNAs in the conversion to psychosis, including a schizophrenia-associated single nucleotide polymorphism in *MIR137HG* that is among the strongest known predictors of age of onset in patients with schizophrenia. Finally, we examine how hemizygosity for *DGCR8*, which encodes an obligate component of the complex that synthesizes miRNA precursors, may contribute to the onset of psychosis in patients with 22q11.2 microdeletions and how animal models of this disorder can help us understand the many roles of miRNAs in the onset of schizophrenia.

## 1. Introduction

MicroRNAs (miRNAs) are strongly implicated in neuronal function. These small noncoding RNAs first bind to mRNA targets containing complementary sequences, and then, they inhibit protein synthesis from the mRNA target or promote target degradation. A single miRNA may regulate hundreds of mRNA targets, and many mRNAs are dynamically regulated by multiple miRNAs. The mammalian brain expresses most of the hundreds of miRNAs that have been identified to date. MiRNAs are intimately intertwined with neurodevelopmental signaling, synaptic plasticity, and adult neuronal activity. Disruptions in the miRNA biogenesis pathway or those in a single miRNA can produce neurologic deficits in human subjects and rodent models. Significant dysregulation of miRNAs and the miRNA biogenesis machinery has been reported in almost every neurodevelopmental, neurodegenerative, or psychiatric disorder in which they have been examined. However, few disorders have received as much attention from miRNA research as schizophrenia (SCZ).

SCZ is a severe psychiatric disorder with a lifetime prevalence of 0.7% in populations worldwide [1]. SCZ symptoms include hallucinations, delusions, disorganized thoughts and speech, diminished or inappropriate emotional expression, apathy, reduced social drive, and deficits in attention and working memory; however, the specific symptoms and their severity vary greatly across patients [2]. SCZ is highly heritable [3], and hundreds of gene variants have been linked to SCZ risk. The disease is also associated with a wide array of environmental factors (e.g., prenatal viral infection, maternal and early life stress, adverse child rearing, and adolescent drug abuse) (reviewed in [4]). SCZ is incurable, chronic, devastating to patients and their families, and one of the most costly mental disorders for national health care systems worldwide [5].

Multiple lines of evidence suggest that the miRNA pathway is central to SCZ. First, postmortem brain studies suggest multiple miRNAs are dysregulated in patients with SCZ [6,7,8,9,10,11,12,13,14]. Second, when compared to other protein-coding genes, SCZ-risk genes contain significantly more predicted miRNA-binding sites [15]. Third, several single nucleotide polymorphisms (SNPs) and variable number tandem repeats near or within *MIR137HG*, the gene that encodes the miRNA miR-137, are highly associated with SCZ risk [16,17,18,19,20,21]. Fourth, brain miRNA levels are sensitive to environmental factors associated with increased SCZ risk. For example, maternal immune activation and adolescent cannabis exposure alter the levels of several miRNAs from the *MIR379/410* gene cluster in the entorhinal cortex of adult rats [22], suggesting that early life events can have long-term effects on brain miRNAs. Finally, the genomes of patients with SCZ are more likely to contain rare copy number variants (CNVs) that overlap with miRNA-encoding genes [23].

**Figure 1:**
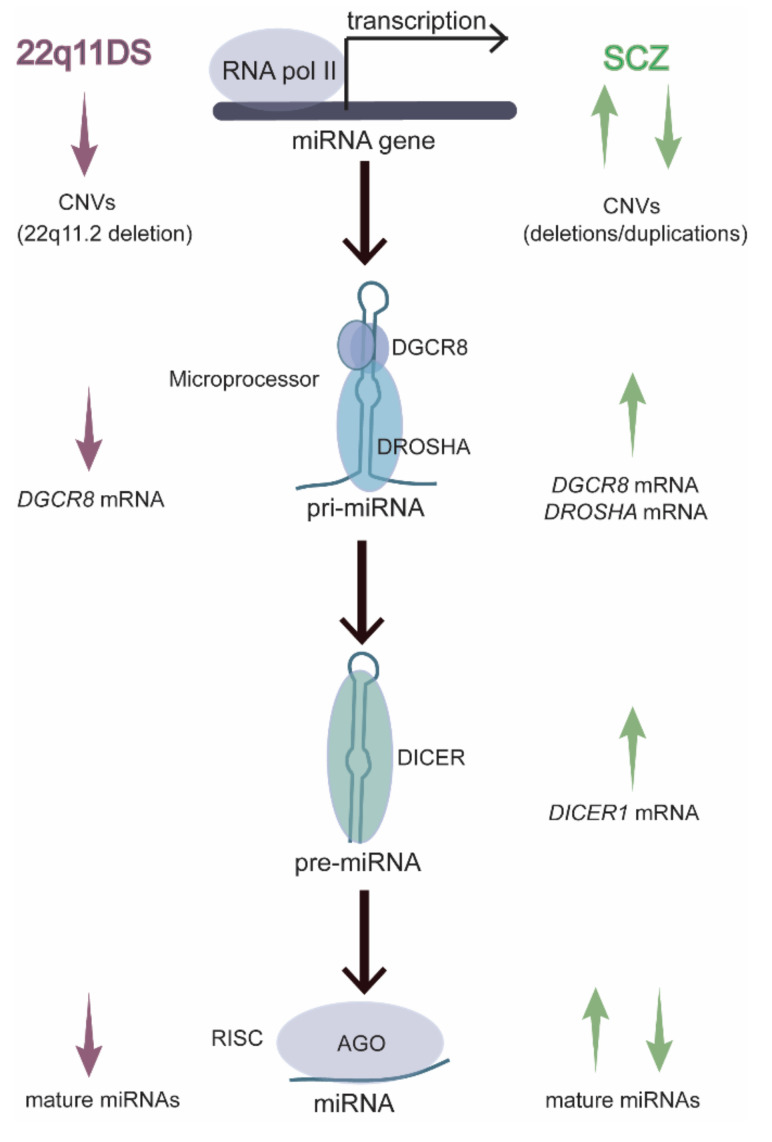
Overview of the miRNA pathway and its dysregulation in 22q11DS and idiopathic SCZ. The miRNA pathway begins when transcription produces a pri-miRNA, which undergoes sequential processing into a pre-miRNA by the Microprocessor and DICER to produce a mature miRNA. The mature miRNA is then loaded into an AGO protein complex to produce an active RNA-silencing complex (RISC), which binds to mRNAs containing complementary sequences and promotes mRNA degradation and/or inhibits protein synthesis. Current models of 22q11DS, which is associated with elevated SCZ risk, suggest that *DGCR8* haploinsufficiency impairs Microprocessor activity, leading to widespread downregulation of miRNAs. In contrast, several studies suggest that several components of the miRNA pathway (i.e., *DGCR8*, *DROSHA*, and *DICER1* mRNAs) are upregulated in idiopathic SCZ. Furthermore, in patients with idiopathic SCZ, overrepresentation of CNVs affecting miRNA genes (outside of the 22q11.2 region) and dysregulated miRNA levels in postmortem brain suggest that both up- and downregulation of miRNAs may contribute to SCZ risk. Abbreviations: AGO, Argonaute protein; CNV, copy number variant; RNA pol II, RNA polymerase II.

Additional lines of evidence suggest that the disruption of protein components in the miRNA biogenesis pathway contributes to SCZ risk. The miRNA is synthesized in a multistep process (Figure 1), beginning with the transcription of a miRNA gene to form a primary miRNA transcript (or pri-miRNA). The pri-miRNA contains one or more hairpin structures that are recognized by the RNA-binding protein DGCR8 and cleaved by the ribonuclease DROSHA to form miRNA precursors (pre-miRNAs) that are exported from the nucleus [24,25,26,27,28,29,30]. Within the cytoplasm, DICER cleaves the pre-miRNA loop and selectively loads one strand into an Argonaute (AGO1-4) protein-containing complex, which mediates the miRNA’s effects on mRNA degradation and translation inhibition [31,32,33,34,35,36].

Dysregulation of miRNA synthesis may increase SCZ risk. Both increases and decreases in the levels of specific miRNAs have been reported in postmortem SCZ brain samples. Hemizygous microdeletions in the 22q11.2 region, CNVs which include the *DGCR8* gene, lead to an ~20- to 25-fold increase in SCZ risk [37,38], and studies in animal models of 22q11.2 deletion syndrome (22q11DS) report widespread deficits in brain miRNA levels [39,40]. By contrast, several reports suggest that *DGCR8*, *DROSHA*, and/or *DICER1* mRNA levels are increased in patients with SCZ but lacking 22q11.2 mutations [7,14,41]. MiRNA levels are tightly controlled in the mammalian brain. Studies in animal models suggest that overexpression or inhibition of miRNAs disrupts neuronal function, so it is quite possible that elevated or reduced activity in the miRNA pathway increases the risk of SCZ.

The roles of miRNAs in SCZ onset are poorly understood. MiRNAs are critical for fetal, infant, and childhood brain development. Other environmental and genetic risk factors associated with SCZ also appear to disrupt early development. However, SCZ onset before puberty is relatively rare, with an estimated prevalence of 1 in 40,000 children younger than 13 years [42]. The rates of psychiatric symptoms increase during puberty and post-pubertal adolescence, including the prodromal symptoms that precede psychosis and the eventual onset of SCZ in some subjects [2]. First-episode psychosis and a SCZ diagnosis typically occur between the ages of 15 (adolescence) and 25 (early adulthood), though some subjects, particularly females, experience onset after age 30 [43]. These studies strongly implicate disruptions in adolescent neurodevelopment in SCZ etiology. However, despite the substantial evidence supporting a role for miRNAs in SCZ etiology, the functions of miRNAs in the adolescent brain remain poorly understood.

In this review, we will examine evidence of miRNAs functioning in the typical maturation of adolescent and early adult brains and in the disrupted developmental trajectories that may underlie SCZ onset. Few studies have directly examined miRNA functions during typical adolescence, and fewer have examined their dysregulation in SCZ model systems. To determine the roles of miRNAs in SCZ onset, we will examine each component of this argument individually. First, the brain undergoes widespread changes during adolescence, and miRNA levels and activity also appear to be dynamic during that time. Next, although we cannot examine miRNA dynamics during SCZ onset in the brains of living patients, evidence from peripheral tissues suggests that miRNA dysregulation occurs during the prodromal phase of disease, as well as after disease onset. Then, we will present a case study of two miRNAs that are highly dynamic during adolescent brain development to explore how miRNA dysregulation during adolescence can interfere with typical development and contribute to disease onset. Finally, we will examine miRNAs and the miRNA pathway components that are strongly associated with SCZ risk but only poorly understood during adolescence and explore how they might contribute to disease onset.

## 2. Temporal Dynamics in the Levels of MiRNAs across the Lifespan

One finding is almost universal among studies examining how miRNA levels vary with age—miRNA levels are highly dynamic throughout the lifespan. A substantial portion of miRNAs are differentially expressed between two or more periods of development or aging (Table 1), and miRNA-profiles become more divergent across brain regions with age [44]. In this section, we will examine how age influences miRNA levels in the mammalian brain, with an emphasis on studies that contain either one or more adolescent time point or time points that span adolescence, thereby allowing changes in miRNA levels during adolescence or early adulthood to be inferred.

Because each miRNA can target hundreds of mRNAs, widespread changes in miRNA levels are predicted to have dramatic effects on cellular function. Several studies have reported global changes in brain miRNA levels associated with age, yet their results have been conflicting. The miRNA microarray studies conducted by Miska et al. [52] and Moreau et al. [47] found that miRNA levels increase with age in mouse and human brain, respectively. Beveridge et al. [48] used Illumina miRNA microarrays to examine miRNA levels in the human dorsolateral prefrontal cortex (DLPFC, Brodman’s area 46) of human subjects, ranging from 2 months to 78 years of age. They found that overall miRNA levels decline with age [48]. Intriguingly, the inflection point at which global miRNA levels switched from an upward to a downward trajectory occurred during late adolescence, at ~17 years of age. However, several of the miRNAs reported by Beveridge et al. to decrease with age (e.g., miR-29a-3p, miR-29c-3p, and miR-132-3p) were reported to increase with age in other studies (Table 2). Differences in study design and methods may explain the discrepancies in these findings. For example, Miska et al. used the first miRNA microarray, which detected far fewer unique miRNAs than later iterations of the method and did not adequately distinguish different miRNAs from the same family [52]. Furthermore, the samples analyzed by Moreau et al. were mostly derived from embryonic or early postnatal timepoints; the only adult-derived samples were commercially available samples, one from an elderly subject (81 years) and a second representing a pooled sample from 23 subjects with an average age of 68 years [47]. Moreau et al., therefore, had poor temporal resolution in childhood through elderly timepoints, and the adult timepoints may have been biased by differences in sample preparation, relative to the younger samples.

More recently, a larger study of 109 subjects used small RNA-seq to examine miRNA levels across the lifespan (second trimester to 78 years of age) in the human DLPFC (BA 46) [11]. Like Beveridge et al., Hu et al. [11] found that most miRNAs peaked in expression before adolescence. A smaller study conducted by Somel et al. [45] similarly found that most major transitions in miRNA expression occur during childhood (~3–4 years) or early adulthood (~20–25 years) [45]. However, that study included only one adolescent (~14 years) and one subject in early adulthood (25 years), so temporal resolution was poor in the indicated age ranges. Consistent with larger studies, Somel et al. found that a few miRNAs exhibit expression patterns that are specific to adult aging, and expression changes associated with aging tend to be extensions or reversals of patterns observed during brain development.

Overall, studies examining miRNA levels across the lifespan have been few in number, have come to sometimes conflicting conclusions, and have lacked consistency in their methods. Despite adolescence being critical in the onset of psychiatric disorders, those time points have been underrepresented in studies examining miRNAs in the brain. However, the following picture has emerged.

During brain development, from the prenatal stage to preadolescence, miRNAs are expressed in waves, with different combinations of miRNAs peaking at different stages. During adolescence and/or early adulthood, miRNA levels become less dynamic, as they transition from their developmental patterns. Most miRNAs are then placed on a trajectory that they will follow for decades during adult aging. This transition period might be particularly vulnerable because deviations from normal patterns might accumulate over time. However, further exploration is needed to understand miRNA dynamics in the human brain during this period, and further research in animal models is needed to understand the long-term impact of disruptions to miRNA levels during this time and whether those disruptions contribute to psychiatric disease.

## 3. Temporal Dynamics in the Activity of MiRNAs across the Lifespan

The activity of a miRNA can change with age in the absence of any change in its levels. Recent evidence demonstrates that miRNA activity also depends on sequence variation, the availability of mRNA target sites within a given cellular context, and the overall state of the protein-synthesis pathway within which the miRNA pathway functions.

MiRNA sequences in vivo often differ from the canonical form listed in miRBase [56,57,58,59,60,61] or other databases. MiRNA microarrays and RT-qPCR assays are typically designed to target the canonical form; thus, they often cannot reliably distinguish between miRNA sequence variants, or “isomiRs”, and they may fail to detect some functional variants entirely ([62], reviewed in [63]). Sequencing of miRNAs can detect isomiRs, but traditional analysis pipelines consider only the sum of a miRNA’s isomiRs rather than how individual isomiRs vary with age or other biological factors.

**Figure 2:**
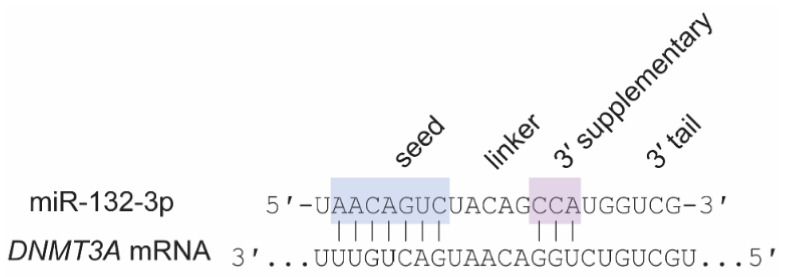
Anatomy of a miRNA. The miR-132-3p canonical sequence targets several sites within the DNMT3A-3′ UTR, including the one shown (interaction predicted by miRbase). Targeting is mostly dictated by the seed sequence (shaded in blue) within the miRNA: nucleotides 2-7 or 2-8 (5′ to 3′). Additional targeting can occur within the 3′ supplementary region (shaded in pink), which is separated from the seed sequence by a noncomplementary linker sequence. The linker sequence varies in length, depending on the target and the position of the 3′ supplementary region. The final nucleotides near the 3′ end of the miRNA form the miRNA’s 3′ tail. The 3′ tail rarely contributes to mRNA targeting but undergoes frequent editing by exonucleases, which trim the miRNA, and nucleotidyl transferases, which add nucleotides to the tail of the miRNA.

The loss or addition of nucleotides at the 5′ end of a miRNA shifts the seed sequence and can have a dramatic impact on miRNA targeting [64,65] (Figure 2). RNA editing in the seed sequence can also alter targeting [66]. Recent research suggests that sequence complementarity closer to the 3′ end of a miRNA (i.e., the 3′ supplementary region) can modulate targeting efficiency [67,68], suggesting that editing in this region also affects targeting, though to a lesser degree. However, most isomiR variation occurs at the terminal 3′ end (i.e., the 3′ tail), leaving the seed and 3′ supplementary regions largely unaffected [69]. In vitro studies suggest that nucleotide loss or addition at the 3′ tail can influence Argonaute protein 2 (AGO2) loading and dissociation [70] and may influence miRNA stability [71,72,73] (but also see [74]). Together, these studies suggest that age-associated variation in the sequences of individual miRNAs significantly influence miRNA activity independent of age-associated variation in miRNA levels.

To date, only one study has examined the effects of age on isomiR variation in the mammalian brain. Juvvuna et al. [75] found that overall miRNA length declines in mouse brain between embryonic day (E) 12.5 and postnatal day (P) 60 (early adulthood) due primarily to increased 3′ trimming of mature miRNAs [75]. Increased 3′ trimming was attributed to an increase in the ratio of AGO2 to AGO1 in the adult brain, though the enzyme responsible for the trimming was not identified. Further research is needed to elucidate the mechanisms underlying increased 3′ trimming; to determine its functional consequences; and to clarify whether it occurs during early development, adolescence, and/or adult aging.

The cellular function of a miRNA is determined almost entirely by the mRNAs that it targets. The mRNA targets available to a miRNA depend on the mRNAs co-expressed with the miRNA within a cell, the subcellular localization of those mRNAs relative to the miRNA, and the accessibility of miRNA-binding sites along the mRNA, all of which may vary with age in ways that are poorly understood. Notably, in most studies, miR-137 levels in the brain are relatively stable with age (Table 2 and Table 3). However, AGO HITS-CLIP (high-throughput sequencing of RNA isolated by crosslinking immunoprecipitation) studies reveal that miR-137 targets largely nonoverlapping mRNAs in the developing human brain (gestation week (GW) 15–16 or GW19–20) [76], relative to adulthood (44–68 years) [77]. This suggests that miR-137 has different functions at different developmental stages.

To date, no HITS-CLIP studies have reported AGO-binding sites within the adolescent or early adult brain in any mammalian brain tissues. Furthermore, most miRNA–mRNA interactions have been validated in cell lines using in vitro reporter systems (e.g., luciferase assays); such systems cannot replicate the effects of age or brain-specific cellular contexts on miRNA targeting. Therefore, to identify miRNA–mRNA target interactions that contribute to adolescent brain maturation and may contribute to age of onset of psychiatric disease, it is crucial to identify the specific miRNA–mRNA target interactions that are present at these timepoints, during both typical development and disease onset.

Finally, several lines of evidence suggest that overall miRNA activity within the brain varies with age. Functionally, miRNAs primarily inhibit protein synthesis, either by directly inhibiting translation or by promoting the degradation of targeted mRNAs. Therefore, age-associated changes in miRNA activity are predicted to cause age-associated changes in protein synthesis. Studies in a variety of model systems suggest that the rates of protein synthesis peak during early postnatal development, before the onset of adolescence, and then decline with age throughout adolescence and adult aging, though the rate of this decline slows with age [84,85,86,87,88] (but also see [89]). This peak in protein synthesis rates also roughly coincides with the inflection point in miRNA levels noted in early childhood/postnatal development by some studies (Table 1), thereby suggesting that tight regulation of mRNA translation is critical to early development. The gradual age-associated decline in protein synthesis in the brain that occurs during adolescence and early adulthood may also implicate changes in miRNA regulation during that time. Theoretically, overall miRNA activity might increase with age, thereby contributing to a decline in protein synthesis. Alternatively, miRNA activity might decline with age due to a decreased need for miRNA regulation. Dysregulated protein synthesis is implicated in SCZ and other adult-onset psychiatric disorders. Olfactory neurosphere–derived cells from patients with SCZ exhibit reduced overall protein synthesis in vitro, relative to cells derived from control subjects [90]. No deficits were observed in fibroblasts, suggesting that the deficit is limited to specific cell types. Similarly, dopaminergic neurons derived from induced pluripotent stem cells from patients with 22q11DS exhibit an ~50% reduction in global protein synthesis, relative to cells from control subjects [91]. Thus, age-associated changes in brain protein synthesis may interact with disease-associated deficits in protein synthesis in patients with SCZ to alter brain function; however, this hypothesis has not been tested in patients or animal models.

The relative importance of miRNAs in regulating gene expression (relative to other methods) may change with age. During preadolescent development, mRNA and protein levels tend to be more highly correlated [92,93], suggesting that transcription drives most changes in gene expression. However, in the human cortex, the correlation between mRNA and protein levels weakens during postadolescent adult aging [92,93], suggesting that posttranscriptional regulation becomes more important with age. At least some of this uncoupling has been attributed to RNA-binding protein–binding sites within the mRNAs [92], thereby implicating age-associated changes in mRNA translation, mRNA stability, and protein synthesis in this uncoupling.

Age-dependent miRNA activity is particularly evident in a group of discordant genes in which the mRNA and protein levels both increase during childhood and adolescence but then diverge during adulthood, with mRNAs continuing to increase but protein levels declining during postadolescent aging [92]. This gene set exhibits evidence of an enrichment in AGO2-binding sites and predicted target sites for miRNAs that are dynamic during aging [45,92,94]. Together, these data suggest that miRNA targeting exerts greater influence over protein levels with increased age, and the brain becomes more sensitive to miRNA dysregulation during the transition from adolescence to adulthood than during earlier stages of development.

In summary, these studies indicate that the miRNA pathway undergoes strong temporal regulation throughout the lifespan, including during adolescence. The levels of at least a subset of miRNAs appear to be highly dynamic during adolescence, and miRNA activity may change independent of miRNA levels. Some of these changes may directly contribute to adolescent brain development, and their dysregulation may cause the onset of psychiatric disease including SCZ. However, further research is needed to fully elucidate the miRNA pathway during this critical period of brain development, during its typical trajectory and during disease onset.

## 4. Neurobiology during the Age of SCZ Onset

**Figure 3:**
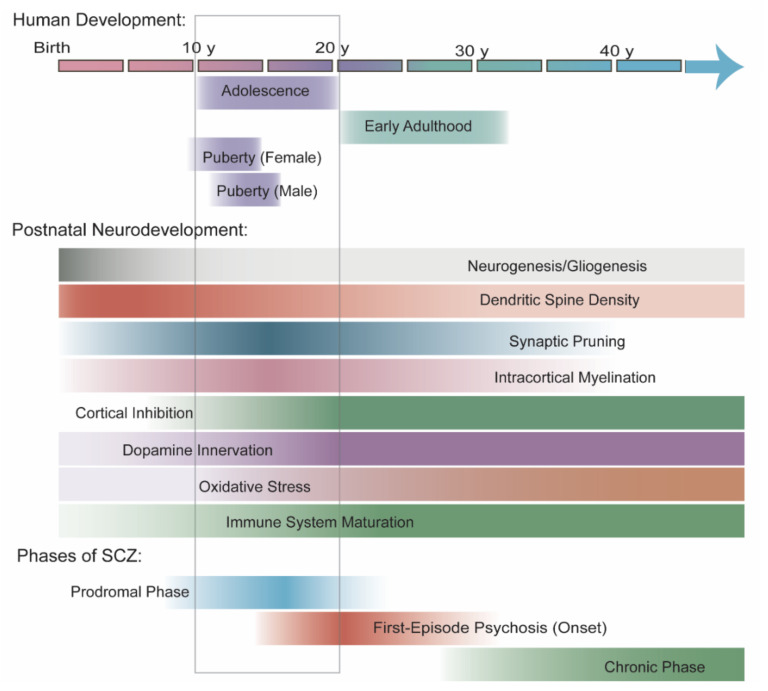
Overview of human postnatal neurodevelopment and the stages of SCZ. The prodromal phase of SCZ typically overlaps with adolescent development, and first-episode psychosis (and diagnosis) typically occurs in late adolescence or early adulthood. The chronic disease phase persists throughout adulthood. The onset of early SCZ symptoms during adolescence overlaps with many facets of brain maturation, including intracortical myelination, synaptic pruning, maturation of GABAergic and dopaminergic signaling in the PFC, increases in oxidative stress markers, and maturation of both the innate and adaptive immune systems. Dysregulation of any (or all) of these processes might contribute to symptom onset.

Adolescence marks the end of childhood, the onset of sexual maturity, and a transition into adulthood. Rapid changes occur in body growth, secondary sexual characteristics, stress response, cognition, and social development through the coordinated action of multiple signaling axes in the adolescent brain. Cognitively, adolescence is marked by increased emotionality and better capacity for executive function and abstract thinking, but also increased vulnerability to stress. Behaviorally, adolescence is marked by not only increased risk taking, exploration, sociability, and independence but also increased susceptibility to drugs of abuse and addiction. Widespread changes in the neural circuity underlie these cognitive and behavioral changes, and disruption of adolescent brain maturation may contribute to the onset of many psychiatric disorders. In SCZ, cognitive, negative, and subthreshold psychotic symptoms often first appear during adolescence (in the prodromal phase) and predict the onset of first-episode psychosis during late adolescence or early adulthood (Figure 3).

MiRNAs are implicated in the control of puberty (i.e., the physical changes during adolescence that lead to sexual and reproductive maturity). Gonadotropin-releasing hormone (GnRH) is essential for puberty onset and adult fertility. In mice, conditional knockout of the *Dicer* gene within hypothalamic neurons causes the progressive loss of GnRH at the onset of adolescence, an almost complete loss of GnRH in adulthood, and infertility [95]. Therefore, miRNAs are essential to the onset and successful completion of puberty. Puberty is also associated with its own neurodevelopmental changes independent of chronological age, both in humans (reviewed in [96]) and animal models (reviewed in [97]). Conversely, adolescent maturation of some neural circuits and behaviors occurs independent of puberty and in the absence of gonadal hormones [98,99,100]. For most adolescent neurodevelopmental processes, however, the relative contributions of puberty versus chronological age remain poorly understood.

Some neurodevelopmental processes typically associated with fetal, infant, or childhood development extend into adolescence and early adulthood. For example, the majority of neurogenesis occurs prior to birth, yet limited neurogenesis continues in the adolescent and adult striatum and hippocampus and may continue in olfactory bulb [101,102,103,104,105,106] (also reviewed in [107]). Similarly, although the majority of gliogenesis occurs prior to birth, new oligodendrocytes and microglia are generated throughout adulthood, and astrocytes continue to proliferate in response to environmental cues [108,109,110,111,112].

Dysregulation of dopamine (DA) signaling underlies psychotic symptoms and may contribute to other SCZ symptoms (reviewed in [113]). Antipsychotics that alleviate positive symptoms of SCZ primarily target D2 DA receptors (DRD2s) [114]. DA signaling within the PFC dramatically changes during adolescence and may directly contribute to SCZ onset. Prior to adolescence, the DRD2 agonist quinpirole has no effect on fast-spiking interneuron excitability. By P50 in rats, DRD2 agonists are strongly excitatory in this cell type [115] and have a net inhibitory effect on pyramidal neuron activity [116]. Overall, DA concentration and innervation peak in the PFC during adolescence in nonhuman primates [117,118]. In mice, DA axons originating in the ventral tegmental area extend from the nucleus accumbens to PFC during adolescence [119], i.e., the only known example of long-range axonal growth during adolescence. In the mouse PFC, miR-218 inhibits DCC [120], a receptor that is critical for correct DA axon targeting [119]. MiRNAs also limit D1 DA receptor, DRD2, and DA transporter levels [121,122,123]; some of this regulation is age-dependent, as we will discuss later in this review. Therefore, miRNAs are essential for shaping DA signaling during adolescence and may contribute to DA dysregulation during SCZ onset in patients through multiple mechanisms.

Markers of oxidative stress increase at the onset of adolescence and during adult aging, both in the brain and periphery [124,125,126]. In brain-derived mitochondria, the rate of aerobic respiration increases during early postnatal and adolescent development, reaching adult levels at ~P60 (end of adolescence/onset of adulthood) in rats [127]. Oxidative stress occurs when the reactive oxygen species formed as natural byproducts of aerobic respiration accumulate to deleterious levels and cause damage to DNA, lipids, and other cellular components susceptible to oxidation. Cells contain endogenous antioxidants (reducing agents), such as glutathione, to maintain redox balance, but reactive oxygen species that accumulate beyond a cell’s capacity to balance them can be particularly detrimental to cells with higher levels of aerobic respiration, such as oligodendrocytes [128,129,130] and fast-spiking parvalbumin-positive (PV^+^) interneurons (reviewed in [131,132]).

Oxidative stress damages oligodendrocytes and disrupts myelination in adolescent mice [130]. Although most long-range axonal projections complete myelination during infancy, many intracortical axons undergo myelination during adolescence or early adulthood [110,133,134,135,136]. MiRNAs are essential for oligodendrocyte maturation and myelination (reviewed in [137]), but research to date has largely focused on their functions in early development or in more severe disruptions observed in demyelinating diseases, e.g., multiple sclerosis.

Like the DA system, GABA signaling within the frontal lobe matures during adolescence in primates [138,139,140,141,142] (reviewed in [143]). Decreases in GABAergic interneuron markers are frequently observed in postmortem SCZ brain and may underlie some cognitive deficits in patients (reviewed in [144]). However, GABA deficiencies are less consistently observed in neuroimaging studies in patients with SCZ [145,146,147]. MiR-101 promotes the maturation of GABA signaling during early development [148] and may be elevated in the DLPFC of patients with SCZ [7]. Later in this review, we will examine a potential role for miR-29a-3p in regulating the maturation of perineuronal nets (PNNs) surrounding cortical PV^+^ interneurons during adolescence [83], which limits some forms of age-dependent plasticity in adulthood. Thus, miRNA dysregulation might disrupt GABA signaling in SCZ through several mechanisms.

Overall synaptic density peaks during early childhood, but synaptogenesis continues in the PFC until mid-adolescence [149]. Synaptic pruning begins in childhood, causing a net decline in synapse numbers that extends through the third decade of life in humans [150,151]. Studies in animal models suggest the rate of pruning may peak in adolescence [152,153,154,155,156]. According to the “Feinberg Hypothesis” [157], excessive synaptic pruning during adolescence may underlie gray matter deficits and reduced dendritic spine densities in patients with SCZ. Although the roles of miRNAs in synapse formation and morphology have been heavily studied, those in synaptic pruning have not been directly examined.

Immune signals influence most aspects of neuronal function. Both the innate and adaptive immune systems mature during adolescence [158,159,160,161] (reviewed in [162,163]). Variation in the major histocompatibility complex immune loci is strongly associated with SCZ [16,18,164,165] and mediated, in part, by the Complement Component 4 genes *C4A* and *C4B* [166]. Both neurons and microglia express components of the complement system [167], which is implicated in microglia-mediated synaptic pruning during adolescence and local inflammatory signaling within the brain [152,168,169,170]. Elevated inflammation has been observed in patients at all stages of SCZ (reviewed in [171]). During adolescence, excess inflammation may cause excess synaptic pruning that disrupts neuronal circuits that are critical for adult cognition and behaviors [172].

Extracellular miRNAs can stimulate inflammation by directly binding to toll-like receptors (TLRs) [173], rather than by binding to mRNA. The significance of this function in adolescence or earlier development remains unknown; however, TLRs and related adapter proteins are highly dynamic during postnatal neurodevelopment (reviewed in [174]) and directly contribute to neurogenesis and other aspects of brain maturation [175,176,177]. Whether miRNA binding to TLRs directly contributes to immune and/or neurodevelopmental dysfunction in SCZ remains unknown. However, extensive cross-communication occurs between TLRs and the complement system–signaling pathways [178], suggesting a novel mechanism by which miRNAs might influence synaptic pruning during adolescence.

In summary, the human brain undergoes widespread changes during adolescence. Some developmental processes (e.g., myelination, synaptic pruning, DA axon outgrowth) converge with other processes that mark a transition into adulthood and adult aging (e.g., increased oxidative stress, immune maturation, and increased inflammation). This convergence coincides with the onset of symptoms in SCZ (Figure 3) and underlies many proposed mechanisms of SCZ pathogenesis. Few studies have examined the roles of miRNAs in these processes during adolescent development or directly implicated miRNAs in their disruption at SCZ onset. However, miRNAs remain particularly attractive candidates for SCZ research because a single miRNA can have a wide variety of functions in adolescent brain development, suggesting that miRNAs influence many aspects of SCZ etiology.

## 5. Peripheral MiRNAs during Conversion to Psychosis

Ideally, miRNA levels could be tracked within brain regions of interest before, during, and after SCZ onset. Using family history and early onset symptoms, we can identify individuals at high risk of psychosis before SCZ onset; however, only 12–35% of individuals at high risk convert to psychosis within 2–2.5 years [179,180,181,182,183] (reviewed in [184]). Although this conversion rate is dramatically higher than that in the general population (~500-fold increase by some estimates [185]), current criteria cannot predict psychosis or SCZ onset with certainty. Consequently, we cannot use postmortem brain samples to identify miRNA changes in the brain that immediately precede symptom onset and are most likely to directly contribute to that onset. In contrast, blood samples can be collected from living subjects at high-risk of SCZ at all stages of disease. Here, we describe recent studies examining miRNAs in peripheral tissues in persons at high risk of psychosis (and SCZ).

Phase II of the North American Prodrome Longitudinal Study (NAPLS-2) assessed symptoms prospectively in participants at clinical high risk (CHR) and in controls over a 5-year period [186]. Approximately 25% of individuals at CHR progressed to psychosis within 2 years [187]. A subset of patients consented to blood collections, from which leukocytes were harvested for miRNA sequencing [188]. Subjects were divided into three groups based on baseline symptoms and diagnoses over the 2-year period: (1) control subjects, (2) subjects at CHR who did not progress to psychosis, and (3) subjects at CHR who progressed to psychosis. Although no single miRNA was identified as significantly different across the groups, subjects who progressed to psychosis differed significantly in the combined levels of five miRNAs: four downregulated miRNAs (miR-941, miR-199a-3p, miR-92a-3p, and miR-31-5p) and one upregulated miRNA (miR-103a-3p) [188]. Furthermore, miRNA levels within the controls and the group at CHR who did not progress were strongly correlated, whereas miRNA levels in those who progressed to psychosis were more weakly correlated [188]. Samples were collected only at baseline; therefore, longitudinal data on symptom-associated changes in miRNA levels are not available.

A later study utilizing the same NAPLS-2 miRNA-seq data set examined the relations between leukocyte miRNAs and gray matter reduction in the superior frontal cortex [155,189]. Although cortical thinning is a normal feature of adolescent brain development, the rate of cortical thinning is accelerated in subjects at CHR who progress to psychosis [189,190,191,192]. A set of nine miRNAs was used to develop a classifier function to predict the rate of cortical thinning in the NAPLS-2 data set: miR-103a-3p, miR-140-3p, miR-142-5p, miR-26b-5p, miR-27b-5p, miR-501-3p, miR-183-5p, miR-339-3p, and miR-193a-5p [155]. Notably, although the same miRNA-seq data were used in both studies, only miR-103a-3p appeared in both the miRNA classifier set associated with conversion to psychosis and the classifier set associated with cortical thinning [155,188]. Neither study found that peripheral miRNA levels are sufficient to predict conversion status, even among individuals at CHR, but together they suggest that miRNA data may be useful in predicting conversion to psychosis and structural phenotypes associated with that conversion.

Although the NAPLS-2 miRNA studies included individuals at CHR who did not progress to psychosis, the investigators did not examine the differences between those subjects and the other groups. Such comparisons might identify miRNAs that contribute to SCZ risk without directly contributing to disease conversion or miRNAs that protect individuals at CHR from disease conversion. To date, no studies have examined peripheral miRNAs specifically in individuals at CHR of SCZ but without disease. One study examined miRNAs in whole blood from individuals with high familial risk of bipolar disorder [193], who also have an elevated risk of SCZ [194,195]. Using RT-qPCR, the authors found that miR-15b, miR-132-3p, and miR-652 are elevated in the blood of subjects at high risk of bipolar disorder, relative to controls. Upregulation of all three miRNAs has also been reported in postmortem SCZ brains [7,9,14].

Finally, miRNA levels may change in the periphery in response to the onset of SCZ. One study examined plasma from patients with first-episode SCZ by using miRNA microarrays and found that miR-223-3p is significantly elevated in patients, relative to age-matched controls [196]. A validation experiment using RT-qPCR found that miR-223-3p is also elevated in plasma from patients with SCZ at later stages of disease. Therefore, miR-223-3p may be a useful peripheral biomarker for SCZ onset. Notably, a separate microarray study found that miR-223-3p is also elevated in postmortem DLPFC of SCZ brains [7], suggesting that miR-223-3p plays a role in disease etiology.

Many studies have examined peripheral miRNAs in individuals with advanced SCZ in hopes of identifying miRNA biomarkers. For example, a large, early study of peripheral blood mononuclear cells observed significant downregulation of 17 miRNAs from the *MIR379*/410 cluster in subjects with SCZ [197]. Studies in animal models suggest miRNAs in this cluster are critical for neuronal function [198,199,200], sensitive to environmental risk factors for SCZ [22], and required for typical sociability [198] and anxiety-like behaviors [201]. Other studies have observed peripheral upregulation of miR-137 [202,203], a miRNA with strong SCZ associations that we will discuss later in this review. These and other biomarker studies have been reviewed extensively elsewhere [202,204,205]. However, miRNA levels in subjects with advanced SCZ may also be altered in response to cellular damage during chronic disease or due to extended use of antipsychotics and/or other prescription drugs. These caveats limit the usefulness of these studies in predicting disease onset or understanding the underlying mechanisms by which miRNAs might contribute to its onset.

Recent work incorporating biomarker information with conventional CHR criteria substantially improved predictions of conversion to psychosis [206]. Additional research is needed to determine whether miRNA data can further improve these predictions. Ultimately, the earlier, more accurate identification of patients who will progress to psychosis will facilitate earlier treatment and interventions. Currently available early interventions improve prognosis for patients [207,208,209], and with continued improvement, future interventions might prevent disease manifestation entirely. This goal can be achieved only with greater understanding of the molecular mechanisms underlying disease onset.

Collectively, these studies suggest that miRNAs in peripheral tissues undergo changes in response to the various stages of SCZ in humans. However, peripheral miRNAs are unlikely to directly contribute to disease onset. Next, we will review work from animal models demonstrating that miRNA dynamics contribute to adolescent brain development.

## 6. MiR-29 and MiR-132-3p in Adolescent Neurodevelopment and Disease

Despite large discrepancies in the overall outcomes of studies examining miRNAs across the lifespan, several miRNAs robustly and consistently increase during postnatal brain maturation: the miR-29 family (primarily miR-29a/b/c-3p) and miR-132-3p (Table 2 and Table 3). We will use these miRNAs as a case study to illustrate how miRNAs are crucial for normal brain development, may be dysregulated in the brains of patients with SCZ, and regulate neurological processes implicated in SCZ etiology. These miRNAs were selected primarily because they are highly represented in the existing research literature, particularly in studies of adolescent brain. However, we acknowledge that future research may reveal other miRNAs that play a greater role in adolescent brain development and SCZ onset.

### 6.1. The MiR-29 Family and MiR-132-3p Levels Increase with Age and May Be Dysregulated in SCZ

The miR-29 family primarily consists of three miRNAs generated from two *MIR29* gene clusters in mammals. These miRNAs are identical in their seed sequences (Table 4) (miRBase [56,57,58,59,60,61]), are enriched in mammalian brain relative to other body tissues, and share largely overlapping expression patterns within the brain [210]. The miR-29 family is implicated in a myriad of roles in brain development and function, including regulating neural progenitor cell proliferation [211], axon branching [80], calcium signaling [212], iron accumulation [213], and neuronal survival [214].

Most studies examining human cortex have found that the level of at least one member of the miR-29 family increases significantly during postnatal development [44,45,47] (but also see [48]). Studies in macaque, pig, rat, and mouse have demonstrated that postnatal miR-29 upregulation with age is highly conserved among mammals [45,49,51,53,54,55,80,81,82,83]. Notably, Swahari et al. [55] examined the levels of miR-29 in mouse cortex at P0–P60 and observed a robust increase in all three family members before the onset of adolescence (P0–P20) and during the transition from early to mid-adolescence (P20–P40). Napoli et al. [83] also observed a robust increase of miR-29a-3p in mouse visual cortex prior to adolescence (P10–P25) and throughout adolescence (P25–P60) [83]. Many other studies found an increase in one or more miR-29 family members between embryonic or early postnatal timepoints and adulthood but lacked adolescent time points; therefore, they could not demonstrate when exactly the increase occurred (Table 2 and Table 3).

The *MIR132/212* gene cluster gives rise primarily to miR-132-3p, miR-212-5p, and miR-212-3p. MiR-132-3p is highly implicated in the regulation of dendritic spine morphology in primary neurons and in adolescent brain. Inhibition of miR-132-3p reduces dendritic spine density and inhibits dendritic spine maturation, without affecting overall dendritic morphology in pyramidal neurons in the mouse visual cortex [215]. In contrast, miR-132-3p mimic increases the proportion of spines with a mushroom (mature) morphology in mouse visual cortex [79]. Reduced dendritic spine density on cortical pyramidal neurons is also a well-documented phenomenon in the postmortem brains of patients with SCZ [216,217,218,219]. MiR-132-3p also regulates fear memory [220,221], learned safety [222], sleep [223], and circadian rhythms [224,225], all of which undergo changes during adolescent development [226,227,228,229,230,231] and are disrupted in SCZ [232,233,234,235]. Reduced levels of miR-132-3p during adolescence or adulthood might contribute to these pathologies in some patients with SCZ.

Human studies using microarrays have yielded conflicting results regarding the effects of age on miR-132-3p in the brain; Beveridge et al. [48] reported an overall decrease in miR-132-3p with age, and Moreau et al. [47] reported an increase between fetal and postnatal development [47,48] (Table 2). Notably, Beveridge et al. also reported a decrease in miR-29a/c-3p levels with age. Studies in rodent models have more consistently reported increased miR-132-3p levels during postnatal brain development: miR-132-3p increases during preadolescent development in the rat cortex and hypothalamus and in mouse PFC and visual cortex (Table 3). Tognini et al. [79] also observed upregulation of miR-132-3p in mouse visual cortex between P7 and P30, with strong upregulation in early adolescence (P20–P30). Whether miR-132-3p undergoes additional upregulation during later stages of adolescence or early adulthood is less clear.

Studies investigating miRNA levels in individuals with SCZ have produced conflicting results on the relations between the miR-29 family and miR-132-3p and disease (Table 5). In most studies, neither miR-29 nor miR-132-3p levels were significantly different in the postmortem brains of persons with SCZ [7,8,9,10,11,12,13]. The few studies that have found differences are not in agreement, with some demonstrating decreased levels and others demonstrating increased levels in persons with SCZ [6,7]. A possible explanation for these conflicting results is the strong relation between the miRNAs and age, as exhibited in Table 2 and noted by Beveridge et al. [7]. Any failure to properly age-match control subjects is likely to substantially affect the results of studies examining these miRNAs.

**Figure 4:**
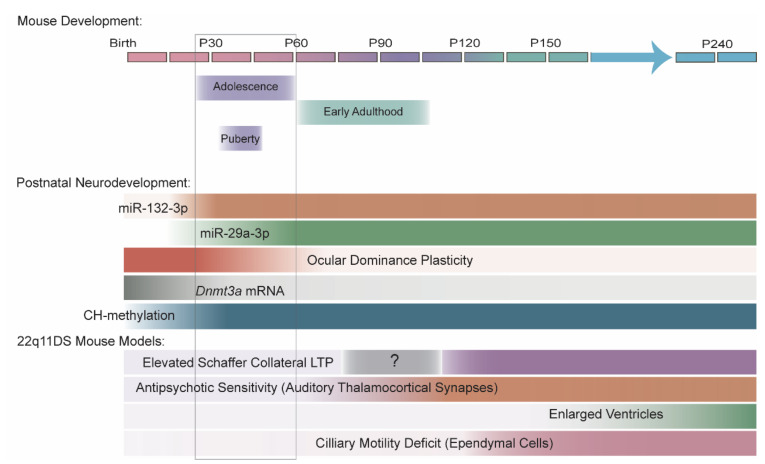
Overview of mouse postnatal neurodevelopment and the timing of SCZ-associated phenotypes in 22q11DS mouse models. Mouse adolescence begins with weaning at ~P22 and ends with the onset of full sexual maturity at ~P60. Increases in miR-29a-3p and miR-132-3p before and during adolescence inhibit ocular dominance plasticity and limit DNMT3A-driven CH-methylation in the visual cortex, providing an example of how temporal regulation of miRNA levels drives late brain maturation in mouse models. Several miRNA-dependent phenotypes also display an age-dependent onset in mouse models of 22q11DS that mimics the developmentally delayed onset of SCZ in patients. In the hippocampus, elevated LTP at Schaffer collateral (CA3–CA1) synapses is absent at P60 but apparent by P120. The exact timing of this phenotype’s onset is unclear, however. Deficits in auditory thalamocortical synapses gradually appear between P60 and P120. Ventricular enlargement is developmentally delayed; however, the ciliary motility deficit in ependymal cells that underlies this phenotype is observed by P120.

In summary, the evidence that miR-29 and miR-132-3p levels are higher in adolescent and adult brain than in childhood brain is robust, though evidence of miR-29 or miR-132-3p dysregulation in SCZ is inconsistent. Further research is needed to fully elucidate the trajectories of both families of miRNAs during typical neurodevelopment and in SCZ. In addition to their individual roles in adolescent brain development, miR-29 and miR-132-3p may also cooperate in regulating processes that are essential for adolescent brain function, specifically the closing of ocular dominance plasticity within the primary visual cortex and the downregulation of *Dnmt3a* mRNA to stabilize CH-methylation in cortical neuron genomes, which we discuss below (Figure 4).

### 6.2. Shared Roles of MiR-29 and MiR-132-3p: Cortical Ocular Dominance Plasticity and DNMT3A

Ocular dominance plasticity is an age-dependent form of cortical plasticity that occurs within the binocular portion of the visual cortex in response to sensory deprivation. If one eye is deprived of sensory input, the neuronal response to visual stimulation of that eye is weakened in the portion of the contralateral visual cortex that receives bilateral visual inputs, and the ocular dominance index (i.e., the relative strength of inputs) shifts toward the ipsilateral, nondeprived eye. Studies in animal models suggest the existence of early critical periods for this form of cortical plasticity: ocular dominance plasticity is strong before adolescence, undergoes an age-dependent decline during adolescence, and is virtually absent in adults [236,237,238,239].

Both miR-132-3p and miR-29a-3p are necessary for ocular dominance plasticity in the mouse primary visual cortex. MiR-29a-3p increases between P10 and P60 in an experience-independent manner [83]. In contrast, miR-132-3p upregulation coincides with the beginning of visual stimulation (i.e., eye opening at ~P13 in mice) [79]. This upregulation continues until early adolescence (~P30) in the presence of normal visual inputs but is substantially blunted if the mice are deprived of visual inputs via dark-rearing [79]. Typically, depriving mice of visual inputs to one eye (i.e., monocular deprivation) in early adolescence for 3–4 days produces a substantial shift in the ocular dominance index toward the nondeprived eye that is detectable at P28. Overexpression of either miR-29a-3p or miR-132-3p in the visual cortex in young mice prematurely blocks ocular dominance plasticity [79,83]. Furthermore, inhibition of miR-29a-3p in adult (~P120) mouse visual cortex restores ocular dominance plasticity that is normally absent by this age [83]. Together, these data suggest that the age-associated increases in miR-29a-3p and miR-132-3p underlie the closing of the critical period for ocular dominance plasticity in the adolescent cortex.

Both Mellios et al. [215] and Tognini et al. [79] attributed miR-132-3p’s role in ocular dominance plasticity to its role in regulating dendritic spine maturation and morphology. In contrast, miR-29a-3p’s role in ocular dominance plasticity has been attributed to its ability to promote maturation of PNNs around PV^+^ interneurons in the mouse visual cortex [83]. These specialized structures of the extracellular matrix limit synaptic plasticity but also protect neurons against oxidative stress [240,241]. Treatment with miR-29a-3p mimics increases PNN intensity during early adolescence, thereby mimicking the mature PNNs that surround neurons in adulthood and reducing plasticity to adult-like levels [83]. In contrast, inhibiting miR-29a-3p in adult mice weakens PNN intensity and reduces sulfonation of chondroitin sulfate position 4, a chemical modification that is typically increased in adult brains relative to adolescent brains. In other words, inhibiting miR-29a-3p in the adult mouse visual cortex partially reverts PNNs to their adolescent state, thereby enabling adolescent-like levels of plasticity.

However, PNNs also protect neurons against oxidative stress, to which fast-spiking PV^+^ interneurons are particularly susceptible. The mature PNNs that surround PV^+^ interneurons in adulthood offer better protection against oxidative stress than do the immature PNNs present in adolescence. Increased oxidative stress and deficits in PV^+^ interneurons and PNN labeling have been documented in patients with SCZ [242,243,244,245,246] and in most SCZ mouse models [247]. Dysregulation of miR-29a-3p, or other miRNAs that regulate PNN integrity, may render PV^+^ interneurons more susceptible to oxidative stress and contribute to PV^+^ interneuron deficits in patients with SCZ.

Both miR-29 and miR-132-3p target the DNA methyltransferase DNMT3A and may contribute to its downregulation during adolescence [10,55]. DNMT3A specifically catalyzes CH-methylation [248], where H indicates T, A, or C nucleotides. In the brain, DNMT3A is predominantly expressed in postmitotic neurons [249]. During early postnatal brain development, neuronal genomes undergo extensive CH-methylation, which ends at ~15 years of age in humans [250] and at ~P21 in mice [249], when DNMT3A levels dramatically decline and miR-29 and miR-132-3p are strongly upregulated. The loss of DNMT3A disrupts early brain development; however, failure to downregulate DNMT3A during adolescence is devastating to adult brain function. The mutation of miR-29–binding sites in *Dnmt3a* in mice leads to elevated DNMT3A protein levels, behavioral abnormalities (e.g., limb clasping), increased seizure susceptibility, and reduced lifespan [55]. *Dnmt3a* mRNA is also upregulated in response to monocular deprivation, and treatment with the DNA methyltransferase inhibitor RG108 blocks ocular dominance plasticity [251], suggesting that *Dnmt3a* downregulation by miR-29 and miR-132-3p contributes to these miRNAs’ roles in ocular dominance plasticity.

Many of the genes downregulated in response to excess CH-methylation are associated with SCZ or other neurologic disorders. Several reports have suggested that *DNMT3A* mRNA is upregulated in the brains of patients with SCZ, though upregulation may be limited to specific neuron subpopulations [252] and/or subjects with chronic (not first-episode) disease [253]. DNMT3A rs2304429 CC genotype may also predict better response to antipsychotics in patients with SCZ [253], and the *DNMT3A* rs2289195 locus is associated with SCZ risk [254]. SCZ-like symptoms (including auditory hallucinations) were also recently reported in three patients with novel, predicted deleterious variants in the *DNMT3A*-coding sequence [255]. Mice hemizygous for *Dnmt3a* also exhibit behavioral deficits, including reduced exploration and increased anxiety-like behaviors [256]. However, no deficits in pre-pulse inhibition (PPI), a common deficit in patients with SCZ, were observed in these mice. Mice with *Dnmt3a* knockout specifically in the forebrain excitatory neurons also exhibit normal PPI and anxiety-like behaviors [257]. However, mice with conditional *Dnmt3a* knockout in inhibitory neurons begin to show behavioral deficits around the time of weaning (~P21) or early adolescence; these deficits include repetitive behaviors, motor deficits, hippocampal learning and memory deficits, and enhanced acoustic startle [258].

In summary, upregulation of miR-132-3p and miR-29 during adolescence is central to the closing of ocular dominance plasticity, regulation of dendritic spine morphology, maturation of PNNs around PV^+^ interneurons, and the downregulation of *Dnmt3a* in adolescent and adult cortex. Dysregulation of either miRNA or their downstream effector DNMT3A disrupts neuronal function in the adolescent brain and may contribute to SCZ onset. However, further research is needed on these miRNAs and their targets in patients with SCZ and mouse models, particularly during adolescent brain maturation.

The cases of miR-29 and miR-132-3p highlight some of the obstacles and opportunities facing miRNA research, particularly as it pertains to adolescent brain maturation and SCZ onset. Research targeting miR-29 demonstrates that the effects of miRNA dysregulation vary with age and that up- or downregulation of the same miRNA at different time points can have very different impacts on neuronal function. Although miRNAs are highly dynamic with age, most studies examine only a few widely spaced time points to draw conclusions about the effects of age on miRNA levels. These studies often completely miss temporary fluxes in miRNA levels and may ignore critical developmental stages, including adolescence. MiRNAs also rarely act alone. Although most functional studies focus on only one miRNA or miRNA family, multiple miRNAs with similar expression patterns may cooperate or compete to regulate the same biological processes and, in some cases, the same mRNA targets. Dysregulation of these targets in patients with SCZ may reflect dysregulation of one or more of these miRNAs, with different miRNAs underlying the same deficit in different patients.

MiR-29 and miR-132-3p are strongly implicated in adolescent brain maturation, but their associations with SCZ remain unclear. In contrast, 22q11.2 microdeletions and genetic variants in *MIR137HG* are strongly associated with SCZ risk and provide more direct evidence that miRNA disruptions underlie this risk, yet how these variants disrupt adolescent brain maturation remains poorly understood. In the following section, we will review recent work suggesting that age-dependent phenotypes in 22q11DS mouse models mimic the age-dependent onset of symptoms in persons with SCZ. We will also review the evidence from human genomic studies suggesting that *MIR137HG* variants predict not only SCZ risk but also variance in age of onset, along with evidence from animal models suggesting how disrupted miR-137 function contributes to SCZ onset.

## 7. 22q11DS Disrupts the MiRNA Pathway in an Age-Dependent Manner

Hemizygous microdeletions in the 22q11.2 region lead to SCZ in approximately one in four patients, representing a 20- to 25-fold increase in risk compared to that in the general population [37,38]. The 22q11.2 region contains *DGCR8*, which encodes a protein component of the Microprocessor (Figure 1), along with several miRNAs, most notably miR-185-5p. The dysregulation of miRNA levels occurs in patients with 22q11DS [259,260], and *Dgcr8* haploinsufficiency impairs miRNA biogenesis in mouse models of 22q11DS [39,40]. The age of onset of SCZ symptoms in patients with 22q11DS is also indistinguishable from that in patients with idiopathic SCZ [261]. Therefore, 22q11DS mouse models provide a valuable tool for investigating the roles of miRNAs in SCZ onset. Of the many phenotypes that have been identified in 22q11DS mouse models, several have a clearly documented age dependence that replicates the timeline of onset in patients with SCZ, and several of the age-dependent phenotypes have been linked to miRNA deficits resulting from *Dgcr8* haploinsufficiency.

Abnormal hippocampal activity may contribute to cognitive symptoms in patients with SCZ [262,263,264,265,266,267]. Our lab previously reported that long-term potentiation (LTP) at hippocampal excitatory synapses between CA3 and CA1 pyramidal neurons (CA3-CA1 synapses) is elevated at ~P120 (mature adulthood) [268] but not at ~P60 (end of adolescence/early adulthood) in 22q11DS mouse models [40]. *Dgcr8* haploinsufficiency is sufficient to replicate both the elevated LTP and its age-dependence. DGCR8 loss leads to reduced hippocampal levels of miR-25-3p and miR-185-5p, which both target *Serca2* mRNA. DGCR8 and miRNA loss leads to an age-dependent increase in synaptic SERCA2, an enzyme that loads Ca^2+^ into the endoplasmic reticulum. Restoration of miR-25-3p or miR-185-5p is sufficient to reduce SERCA2 to wild-type levels and restore LTP in 22q11DS mouse models. Notably, elevated SERCA2 was observed in the PFC and hippocampus of patients with SCZ [40]. The *ATP2A2* locus, the human homologue of *Serca2*, was also identified as one of 108 loci meeting genome-wide significance for association with SCZ by the Schizophrenia Working Group of the Psychiatric Genomics Consortium [18].

Auditory hallucinations are one of the most prevalent symptoms in SCZ, occurring in as many as 80% of patients (though prevalence varies by culture) [269,270]. The onset of hallucinations and other psychotic symptoms typically marks the end of the prodromal phase and the onset of early SCZ disease. Our lab has found that *Dgcr8* haploinsufficiency underlies two age-dependent deficits in 22q11DS mouse models that may be related to auditory hallucinations in patients: behavioral deficits in PPI in an acoustic startle reflex and synaptic deficits in thalamocortical excitatory projections from the medial geniculate nucleus (i.e., auditory thalamus) to the primary auditory cortex [271]. Both deficits are absent at P60 but are present by P120 in mice, suggesting an early adult onset typical of hallucination onset in patients [121]. Both deficits are also sensitive to antipsychotics, the typical therapy for hallucinations and other positive symptoms of SCZ.

Additionally, our lab found that DGCR8 loss in mice leads to reduced levels of miR-338-3p in the auditory thalamus, which in turn leads to elevated levels of DRD2s (encoded by *Drd2*) [121]. Restoration of miR-338-3p or *Drd2* to wild-type levels is sufficient to rescue behavioral and synaptic deficits in *Dgcr8*-deficient mice. Remarkably, *Mir338* haploinsufficiency alone removes the age-dependence of some of these deficits; behavioral deficits and antipsychotic sensitivity are present by P45 (adolescence). This suggests that partial loss of a single miRNA underlies thalamocortical deficits in 22q11DS mouse models. Notably, our lab also observed reduced miR-338-3p levels in auditory thalamus samples from patients with SCZ, relative to age-matched control subjects, though the sample size for this experiment was fairly small (*n* = 7 per group). Finally, like *ATP2A2*, *DRD2* is one of 108 loci that meets genome-wide significance for association with SCZ, per the Schizophrenia Working Group [18]. This finding suggests that the disruption of miR-338-3p targeting of *DRD2* mRNA contributes to thalamocortical disruptions in patients with SCZ.

Together, these studies demonstrate that miRNA deficits underlie multiple synaptic or behavioral phenotypes with age-dependent, early adult onsets that replicate the onset of SCZ in patients with 22q11DS. However, DGCR8 deficiency is present throughout life, raising the question of why these deficits arise during late brain maturation rather than earlier in development. Our lab’s work with miR-338-3p revealed that miR-338-3p levels in the auditory thalamus decline between P60 and P120, independent of genotype [121]. This age-dependent decline, in combination with miR-338-3p loss due to DGCR8 deficiency, may cause miR-338-3p levels to drop below a critical level necessary for DRD2 regulation, allowing the emergence of synaptic and behavioral deficits.

The age-dependent decline in miR-338-3p is not dramatic (only ~20%). As previously discussed, the results of studies examining temporal regulation of miRNA levels are often poorly replicated, even within a single model system and for miRNAs that undergo several-fold increases or decreases in their levels. Furthermore, no mechanism has been proposed for this apparent decline in miR-338-3p levels, and no decline has been reported for miR-25-3p or miR-185-5p in the hippocampus to explain their age-dependent regulation of SERCA2 [40]. Further examination of miRNA levels and activity during adolescence and early adulthood in humans and mouse models is needed to elucidate how they contribute to SCZ onset.

The developmental trajectories observed in mice do not always model those observed in patients. Ventricular enlargement is one of the most replicated structural defects observed in the brains of patients with SCZ [272,273,274]. Our lab found that *Dgcr8* haploinsufficiency underlies enlargement of the lateral and third ventricles in 22q11DS mouse models through a miRNA-dependent mechanism [122]. Although this phenotype is age-dependent, its onset occurs significantly later than that observed in patients. Specifically, ventricular enlargement is present in 8-month-old mice; at 4 months, the ventricles of 22q11DS mice are indistinguishable from those of wild-type mice. In contrast, ventricular enlargement has been observed in patients at SCZ onset, and it progresses with age [275,276,277,278,279].

Therefore, mouse models of 22q11DS imperfectly model the age dependence of SCZ pathologies, with some phenotypes mimicking onset in patients and others following a delayed trajectory. Ventricular enlargement in 22q11DS mice has been attributed to reduced ciliary motility in ependymal cells in the ventricle walls, which may lead to a slow, progressive accumulation of cerebrospinal fluid [122]. This defect in ciliary motility is present by ~P120 in mice, similar to other *Dgcr8-* and miRNA-dependent phenotypes. Therefore, the ventricular enlargement is developmentally delayed in mice relative to humans, but the underlying molecular mechanisms contributing to it may be initiated by developmental events that are more closely aligned across species.

Our lab’s work examining miRNAs in ventricular enlargement highlights an additional obstacle to using 22q11DS mouse models––species differences in the miRNA pathway. In 22q11DS mouse models, decreased levels of miR-382-3p and miR-674-3p lead to elevated D1 dopamine receptors, reduced ciliary motility, and enlarged ventricles [122]. However, the sequence of murine miR-382 listed in miRBase is shifted toward the 3ʹ end of pre–miR-382, relative to the human form, which would alter the seed sequence and could dramatically alter mRNA targeting (Table 4). Furthermore, mouse miR-674-3p appears to have no human ortholog, so this miRNA cannot contribute to ventricular enlargement in patients with SCZ. Conversely, mouse models cannot model the effects of human- or primate-specific miRNAs, some of which are crucial for human brain development [76,280,281] and/or dysregulated in postmortem SCZ brain [282]. Therefore, the specific miRNAs that underlie phenotypes in mouse models may play no role in the SCZ symptoms that they model. Nonetheless, in most cases, miRNAs critical for mouse brain function and dysregulated in SCZ mouse models are highly conserved between mice and humans (Table 4).

Despite these limitations, mouse models of 22q11DS provide a valuable tool for identifying novel miRNAs with potential relevance to SCZ onset, identifying novel miRNA-dependent pathways that underlie SCZ-associated phenotypes, and investigating basic mechanisms by which miRNAs contribute to adolescent and early adult brain maturation.

## 8. *MIR137HG* Variance Predicts Variance in the Age at SCZ Onset

The age of onset of psychosis in patients with SCZ is highly variable. Onset typically occurs between 15 and 35 years of age, and male patients typically have an earlier onset than females [43]. One of the best-known predictors of age of onset occurs at the rs1625579 SNP near *MIR137HG*. In subjects with SCZ, Lett et al. [283] found that the mean age of onset for those with the TT genotype at rs1625579 was 20.8 ± 5.7 years, whereas that for those carrying a protective G allele (GT/GG genotype) was 23.7 ± 9.1 years. In patients with SCZ, the T allele is also associated with larger left ventricle volume, smaller hippocampal volume, and reduced white matter integrity [283] (also see [284]) and may predict more severe negative symptoms and worse cognitive performance [285] (also see [286]).

The rs1625579 SNP was first identified in a SCZ genome-wide association study [16]. The T allele was identified as the major allele and the risk allele for SCZ, while the minor G allele appeared to be protective. Later studies identified additional SNPs in *MIR137HG* and in predicted and validated miR-137 targets that display significant enrichment in the genomes of patients with SCZ [17,18,19,20]. Furthermore, common genetic variants implicated in SCZ are enriched for predicted miR-137–binding sites [15]. Together, these studies strongly implicate miR-137 in genetic risk for SCZ.

Experiments in several cell culture systems suggest that the protective alleles at multiple *MIR137HG* loci are associated with elevated miR-137 levels [20,21,287,288], and some evidence suggests miR-137-3p levels are lower in the brains of neurotypical subjects homozygous for the risk allele (TT) at rs1625579 [289]. Together, these studies suggest that elevated miR-137 is protective against SCZ and delays its onset among patients. However, miR-137 levels may be higher in the peripheral blood of subjects with SCZ [203], and postmortem brain studies have mostly found no evidence of miR-137 dysregulation in SCZ when *MIR137HG* genetic variants are ignored (Table 5). Furthermore, the high frequency of the risk allele among most populations, the low prevalence of SCZ in most populations, and the lack of evidence of miR-137 dysregulation in most patients with SCZ suggest that miR-137 dysregulation alone is not sufficient to promote SCZ onset.

Experiments in mouse models suggest that overexpression and inhibition of miR-137 are disruptive to neuronal function. Mice hemizygous for neuronal *Mir137* exhibit deficits in synaptic plasticity, social behavior, and learning, along with repetitive behaviors [290]. Similarly, hemizygous microdeletions in the 1p21.3 region, which contains *MIR137HG*, are associated with autism spectrum disorders and intellectual disability in human subjects [291,292,293,294,295]. In mice, overexpression of miR-137 in neurons impairs dendrite outgrowth, hippocampal learning, PPI, social behavior, and performance in the novel object-recognition task [287,296,297]. These studies demonstrate that both up- and downregulation of miR-137 disrupt neurodevelopment and suggest that either might contribute to neurodevelopmental disorders.

MiR-137 regulates many neuronal targets that are implicated in SCZ, including several of the 108 loci identified by the Schizophrenia Working Group [18]. Many miR-137 targets are involved in signaling pathways that are central to early neurodevelopment and/or synaptic plasticity, including: neuregulin signaling [298], PI3K–mTOR signaling [298], PKA signaling [290], and glucocorticoid signaling [299]. MiR-137 is also intimately involved in glutamatergic synaptic transmission, as it regulates many presynaptic targets involved in neurotransmitter release and multiple glutamatergic receptors [287,300], including AMPA and NMDA receptor subunits GluA1 and GluN2A, respectively [301,302]. NMDA receptor antagonists mimic positive and negative symptoms of SCZ [303,304,305], and altered DA signaling might arise as a result of dysregulated NMDA receptor activity [113]. Therefore, miR-137′s roles in regulating glutamate signaling may be particularly relevant to SCZ onset.

Although miR-137 regulates neurodevelopment and appears to contribute to SCZ onset, it is unclear how miR-137 contributes to adolescent brain maturation. Several studies suggest that miR-137 levels are highest and most dynamic during perinatal and early postnatal brain maturation and remain high but relatively stable during adolescence and adulthood (Table 2 and Table 3). However, HITS-CLIP studies suggest that miR-137 targeting varies with age [76,77], which suggests that its functions are also age dependent, but those functions during adolescence have yet to be examined.

MiR-137 dysregulation alone is highly unlikely to cause SCZ, but miR-137 may influence how the brain responds to other genetic and environmental risk factors for SCZ. A few studies suggest that miR-137 is protective against oxidative stress and inflammation [306,307], so lower levels of miR-137 might increase susceptibility to those stresses during adolescence. In mouse models, glucocorticoid signaling mediates interactions between genetic risk and adolescent stress that disrupt DA signaling and lead to deficits in PPI and other behaviors during adulthood [308]. MiR-137 targets many components of glucocorticoid signaling, suggesting miR-137 dysregulation could also increase susceptibility to adolescent stress. However, miR-137 function has not been examined within the context of SCZ mouse models, so it is unclear how miR-137 interacts with other genetic or environmental risk factors for SCZ in an adolescent brain.

## 9. Possible Mechanisms of MiRNA-Dependent Onset of SCZ

Although the miRNA pathway is strongly implicated in SCZ, the precise mechanisms by which miRNA dysregulation contributes to disease onset remain poorly understood, in part, because the mechanisms by which miRNAs contribute to typical brain maturation remain poorly understood, especially during adolescence. However, research demonstrates that miRNAs are highly dynamic during all stages of brain maturation and are essential for developmental processes implicated in SCZ onset.

SCZ is a highly heterogeneous disorder (and possibly an umbrella term for several disorders), with an array of genetic and environmental risk factors implicated in its etiology. Research in the 22q11DS field strongly implicates the miRNA pathway, but most studies of miRNA dysregulation in SCZ have failed to implicate the same set of miRNAs. Furthermore, as observed with miR-29 and miR-132-3p, studies often come to opposite conclusions about specific miRNAs. Perhaps the field has been too focused on specific miRNAs, when it is highly likely that no individual miRNA underlies SCZ onset or any aspect of its pathology in all patients.

Heterogeneity in miRNA dysregulation might contribute to heterogeneity of symptoms and their onset times in patients with SCZ. Because different miRNAs perform different functions, they might contribute to SCZ onset by different mechanisms. We propose the following mechanistic categories by which miRNAs might contribute to SCZ onset.

### 9.1. Direct Mechanism: Aberrations in MiRNAs during Adolescence Disrupt Adolescent Brain Maturation and Cause Disease Onset

We propose that the failure to correctly up- or downregulate miRNAs during adolescence will specifically disrupt adolescent brain development and directly contribute to SCZ onset. In this mechanism, early development may proceed as normal, when the miRNAs of interest are either nonessential or expressed at normal levels for early developmental stages. During adolescence, the miRNAs are incorrectly expressed, leading to dysregulation of their targets and disruption of neurodevelopment. For example, both miR-29 and miR-132-3p downregulate DNMT3A during adolescence and adulthood [10,55], but neither miRNA is essential for regulating DNMT3A levels during early postnatal development, when they are virtually undetectable. Genetic or environmental factors that inhibit miR-29 and miR-132-3p most likely have no effect on DNMT3A levels during early development because these miRNAs are not essential during that time. During adolescence, disruptions to miR-29 and miR-132-3p might cause an upregulation of DNMT3A; excess CH-methylation; and age-dependent, late-onset disruptions in neuronal function.

### 9.2. Delayed Mechanism: Aberrations in MiRNAs during Early Development Disrupt Brain Maturation during Adolescence 

This mechanism is most closely related to the “two-hit” model of SCZ [309]. Here, dysregulation of miRNAs might provide the first hit to neurodevelopment, while the second occurs during adolescence and promotes SCZ onset. The second hit might be an additional disruption to neurodevelopment or a normal developmental process that reveals the effects of the first hit.

The Delayed Mechanism might underlie miR-137′s role in SCZ onset. MiR-137 levels are most dynamic in the brain during early postnatal development (Table 2 and Table 3), and miR-137 is implicated in many signaling pathways that are essential to early brain development and implicated in SCZ risk. Small disruptions in miR-137 early in development might not immediately affect neuronal function, but large disruptions might lead to disorders with earlier onset, e.g., autism spectrum disorders or intellectual disability [290,291]. However, small disruptions might render the brain more vulnerable to additional stress during adolescence. This mechanism is particularly attractive for explaining miR-137′s role, because this miRNA may regulate DA signaling [123], which matures during adolescence [310], and cellular responses to oxidative stress and inflammation [306,307]. Disruptions to miR-137 might, therefore, provide “hits” to both early and adolescent development. However, miR-137′s functions within the adolescent brain remain poorly understood.

In adulthood, the brain may become more dependent on posttranscriptional mechanisms of regulation, including miRNAs [92,93]. This mechanism might underlie several miRNA-associated deficits in 22q11DS mouse models [40,121,271]. As previously discussed, deficiencies in DGCR8 and miRNAs are present early in development, but several miRNA-dependent phenotypes do not emerge until early adulthood. Prior to adolescence, small disruptions to the miRNA pathway (the first “hit”) might be insufficient to disrupt the processes that they regulate, perhaps due to compensatory mechanisms that affect transcription. With age, cells may become more dependent on miRNAs, effectively providing a second “hit” to miRNA-dependent processes.

### 9.3. Progressive Mechanism: Aberrations in Individual MiRNAs Cause Small Deficits That Accumulate during Development and Become Disruptive during Adolescence 

Some disruptions in brain function accumulate over time, before they cause detectable deficits. This is consistent with the observation that some SCZ phenotypes progress with age. However, few studies have directly examined how miRNA dysregulation contributes to this progression.

In patients with SCZ, ventricle enlargement is present during the prodromal phase [189] and first-episode psychosis [275,277], and it progresses with age through the chronic disease phase [278,279]. We previously described how deficiencies in DGCR8 and miRNAs contribute to ciliary deficits in ependymal cells and underlie enlarged ventricles in a 22q11DS mouse model [122]. DGCR8 deficiency is present throughout development. This ciliary deficit is detectable by ~P120, suggesting the miRNA deficit is also present by P120, though it is unclear whether the miRNAs underlying this deficit are regulated with age. By contrast, ventricular enlargement appears to be slow, progressive, and undetectable until ~P240. Thus, ventricular enlargement is developmentally delayed in these mice, suggesting that developmental stage may be less important than the duration of time following the onset of the miRNA deficit for this particular structural phenotype.

## 10. Conclusions

We speculate that the direct, delayed, and progressive mechanisms that we propose here contribute to SCZ onset, though different miRNAs may contribute to SCZ onset in different subsets of patients. Additional research is needed to fully elucidate miRNA trajectories during typical neurodevelopment and to determine normal levels of variance across neurotypical subjects. This will set the stage for comprehensive examination of miRNA deviations at different stages of development and their contribution to SCZ onset. We predict that a greater understanding of the molecular mechanisms underlying SCZ onset will facilitate the identification of at-risk subjects for earlier therapeutic intervention, provide novel targets for therapeutic interventions, and, ultimately, position us to delay or even prevent SCZ symptoms entirely.

## Figures and Tables

**Table 1:** Global patterns in miRNA levels with age.

Species	Reference	Region	Method	Age Range	Adolescent Time Points?	Global Patterns in miRNA Levels
Human	Somel et al., 2010 [45]	Superior frontal gyrus of PFC	Small RNA-seq	0–98 y	Yes (one 13 y)	Highly dynamic with age, with inflection points at ~4 y and ~20 y
	Somel et al., 2011 [46]	PFC and cerebellum	Microarray	0–98 y	No	None noted
	Moreau et al., 2013 [47]	Cerebrum	Microarray	GW14–adult ^a^	No	Increase with age
	Beveridge et al., 2014 [48]	DLPFC (BA 46)	Microarray	2 mo–78 y	Yes	Decrease with age, with inflection point at late adolescence
	Ziats and Rennert 2014 [44]	OPFC, DLPFC, MPFC, VLPFC, hippocampus and cerebellum	Small RNA-seq	4 mo–19 y	Yes	Most dynamic during transition from infancy to early childhood
	Hu et al., 2019 [11]	DLPFC (BA 46)	Small RNA-seq	Second trimester–74 y	Yes	Most dynamic/peak expression before puberty
Pig	Podolska et al., 2011 [49]	Cortex and cerebellum	Microarray	F50–3 mo	Yes (3 mo)	None noted
Rat	Krichevsky et al., 2003 [50]	Forebrain	Array ^b^	E12–adult	No	Highly dynamic with age
	Yao et al., 2012 [51]	Dorsolateral cortex	Small RNA-seq	E17–P28	Yes (P28)	Highly dynamic across most time points
Mouse	Miska et al., 2004 [52]	Whole brain	Microarray ^c^	E12.5–4 mo	No	Increase with age
	Eda et al., 2011 [53]	Cerebrum, cerebellum, hippocampus	Microarray	E16.5–19 mo	Yes (1 mo)	Most dynamic in early postnatal brain (P6–1 mo)
	Fertuzinhos et al., 2014 [54]	Primary somatosensory cortex	Small RNA-seq	P4–P180	No	None noted
	Swahari et al., 2021 [55]	Cerebellum	Small RNA-seq	P18 and P250	No	None noted

^a^ Adult samples were commercially derived and elderly. ^b^ First microRNA array consisted of a nylon membrane spotted with oligos. ^c^ First miRNA microarray. Abbreviations: BA, Brodmann’s area; DLPFC, dorsolateral prefrontal cortex; F, fetal day; GW, gestational week; MPFC, medial prefrontal cortex; OFPC, orbitofrontal prefrontal cortex; PFC, prefrontal cortex; VLPFC, ventrolateral prefrontal cortex.

**Table 2:** Changes in specific miRNAs with age in primate and pig brains.

Study Information						miR-29a/b/c-3p		miR-132-3p		miR-137-3p	
Species	Reference	Method	*p*-Value	Age Range	Region	Direction	Differential Age Range	Direction	Differential Age Range	Direction	Differential Age Range
Human	Somel et al., 2010 [45]	Small RNA-seq	Yes	2 d–98 y	Superior frontal gyrus (BA9)	Increased (a) NR (b/c)	2 d–98 y (a)	NR	--	NR	--
	Moreau et al., 2013 [47]	Microarray	No	fetal (GW14–GW24), early postnatal (5 d–4 y), adult *	Cerebrum	Increased (a) Increased (b) NR (c)	Postnatal-adult (a) postnatal (5 d–4 y) (b)	Increased	Fetal-postnatal	Increased	Fetal (GW14–GW20)
	Beveridge et al., 2014 [48]	Microarray	Yes	2 mo–78 y	DLPFC (BA46)	Decreased (a/c) NR (b)	2 mo–78 y	Decreased	2 mo–78 y	Decreased	2 mo–78 y
	Ziats and Rennert 2014 [44]	Small RNA-seq	Yes	4 mo–19 y	DLPFC (BA9, 46)	Increased (b) NS (a/c)	Infancy-early childhood (b)	NS	--	NS	--
					cerebellum (CBC)	NS	--	NS	--	Decreased	Infancy-early childhood
					OPFC (BA11), MPFC (BA32-34), VLPFC (BA44-45), hippocampus	NS	--	NS	--	NS	--
Macaque	Somel et al., 2010 [45]	Small RNA-seq	Yes	16 d–28 y	Superior frontal gyrus (cortex)	Increased (a) NR (b/c)	16 d–28 y (a)	NR	--	NR	--
Pig	Podolska et al., 2011 [49]	Microarray	No	F50, F100, 3 mo	Cortex	Increased (a/b/c)	F100–3 mo	NR	--	NR	--
					Cerebellum	Increased (a/b/c)	F100–3 mo	NR	--	NR	--

* Adult samples were commercially derived and elderly. (a/b/c) indicates specific miR-29 variant reported in each study. Abbreviations: BA, Brodmann’s area; DLPFC, dorsolateral prefrontal cortex; GW, gestational weeks; MPFC, medial prefrontal cortex; NE, not examined; NR, not reported (unclear if NE or NS); NS, not significant; OFPC, orbitofrontal prefrontal cortex; PFC, prefrontal cortex; VLPFC, ventrolateral prefrontal cortex.

**Table 3:** Changes in specific miRNAs with age in rodent brain.

Study Information						miR-29a/b/c-3p		miR-132-3p		miR-137-3p	
Species	Reference	Method	*p*-Value	Age Range	Region	Direction	Differential Age Range	Direction	Differential Age Range	Direction	Differential Age Range
Rat	Yao et al., 2012 [51]	Small RNA-seq	No	E17–P28	Whole cortex (E10 and E13) dorsolateral cortex (E17–P28)	Increased (a/b/c)	P3–P28	Increased	P3–P28	Peak at P0	Increased (E10–P0) Decreased (P0–P28)
	Sangiao-Alvarellos et al., 2013 [78]	RT-qPCR	Yes	P1–adult (>P75)	Hypothalamus	NE	—	Increased	Neonatal-juvenile	NE	—
Mouse	Eda et al., 2011 [53]	Microarray	No	E16.5–19 mo	Cerebrum	Increased (a/b/c)	E16.5–3 mo	Increased	E16.5–1 mo	Increased	E16.5–P6
				P2–19 mo	Hippocampus	Increased (a/b/c)	P2–6 mo	Increased	P2–6 mo	ND	—
				E16.5–19 mo	Cerebellum	ND	—	Increased	E16.5–1 mo	Two peaks	Increased (E16.5–P2) Decreased (P2–6 mo), Increased (6 mo–19 mo)
	Tognini et al., 2011 [79]	RT-qPCR	Yes	P7–P35	Visual cortex	NE	—	Increased	P7–P30	NE	—
	Miller et al., 2012 [10]	RT-qPCR	Yes	E15–P60	PFC	NE	—	Increased	P7–P28	NE	—
	Fertuzinhos et al., 2014 [54]	Small RNA-seq	Yes	P4–P180	S1 cortex	Increased (a) ND (b/c)	P4–P180	Increased	P4–P180	ND	—
	Li et al., 2014 [80]	Microarray	No	E12.5–E18.5, P60	Cortex	Increased (a) ND (b/c)	E18.5–P60	Increased	E12.5–P60	NR	—
		RT-qPCR	Yes	E12.5–P60	Cortex	Increased (a) NE (b/c)	P1–P60 (a)	NE	—	NE	—
				E18.5–P60	Hippocampus	Increased (a) NE (b/c)	P1–P60 (a)	NE	—	NE	—
	Mazziotti et al., 2017 [81]	Small RNA-seq	Yes	P10, P28	V1 cortex	Increased (a/c), NS (b)	P10–P28	Increased	P10–P28	NS	—
	Li et al., 2019 [82]	RT-qPCR	Yes	P10–P120	Hypothalamus	Increased (a/b/c)	P10–P120	NE	—	NE	—
	Swahari et al., 2021 [55]	RT-qPCR	No	P0–P60	Cortex	Increased (a/b/c)	P0–P40 (a/c), P0–P60 (b)	NE	—	NE	—
					Cerebellum	Increased (b) NE (a/c)	P0–P60 (b)	NE	—	NE	—
		Small RNA-seq	No	P18, P250	Cerebellum	Increased (a/b/c)	P18–P250	NR	—	Increased	P18–P250
	Napoli et al., 2020 [83]	RT-qPCR	Yes	P10–P200	Visual cortex	Increased (a) NE (b/c)	P10–P60	NE	—	NE	—

(a/b/c) indicates specific miR-29 variant reported in each study. Abbreviations: F, fetal day; NE, not examined; NR, not reported (unclear if NE or NS); NS, not significant; S1, primary somatosensory; V1, primary visual.

**Table 4:** MiRNA sequences of interest.

MiRNA	Sequence (Human/hsa-)	Sequence (Mouse/mmu-)	Match ^a^	Targets of Interest
miR-25-3p	CAUUGCACUUGUCUCGGUCUGA	CAUUGCACUUGUCUCGGUCUGA	Yes	*ATP2A2* (*Serca2*)^b^
miR-29a-3p	UAGCACCAUCUGAAAUCGGUUA	UAGCACCAUCUGAAAUCGGUUA	Yes	*DNMT3A*
miR-29b-3p	UAGCACCAUUUGAAAUCAGUGUU	UAGCACCAUUUGAAAUCAGUGUU	Yes	*DNMT3A*
miR-29c-3p	UAGCACCAUUUGAAAUCGGUUA	UAGCACCAUUUGAAAUCGGUUA	Yes	*DNMT3A*
miR-132-3p	UAACAGUCUACAGCCAUGGUCG	UAACAGUCUACAGCCAUGGUCG	Yes	*DNMT3A*
miR-137-3p^b^	UUAUUGCUUAAGAAUACGCGUAG	UUAUUGCUUAAGAAUACGCGUAG	Yes	*GRIA1*^b^, *GRIN2A*^b^
miR-185-5p	UGGAGAGAAAGGCAGUUCCUGA	UGGAGAGAAAGGCAGUUCCUGA	Yes	*ATP2A2* (*Serca2*)^b^
miR-338-3p	UCCAGCAUCAGUGAUUUUGUUG	UCCAGCAUCAGUGAUUUUGUUG	Yes	*DRD2* ^b^
miR-382-3p	AAUCAUUCACGGACAACACUU	UCAUUCACGGACAACACUUUUU	No	*DRD1*
miR-674-3p	no human ortholog	CACAGCUCCCAUCUCAGAACAA	No	*DRD1*

The seed sequence is underlined in each miRNA sequence. ^a^ Yes: indicates miRNA sequence is identical in human and mouse; No: indicates species difference. ^b^ Ripke et al., 2014: 108 loci associated with SCZ.

**Table 5:** MiRNA levels in postmortem brains from patients with SCZ.

Brain Region	Reference	Method	miR-29a/b/c-3p	miR-132-3p	miR-137-3p
DLPFC (BA9)	Perkins et al., 2007 [6]	Microarray, RT-qPCR (miR-29b)	Decreased (a/b/c)	NS	NS
	Beveridge et al., 2010 [7]	Microarray	Increased (c) NS (a/b)	NS	NR
	Moreau et al., 2011 [8]	RT-qPCR	NS	NS	NE
DLPFC (BA46)	Kim et al., 2010 [9]	RT-qPCR	NS	Increased	NS
	Miller et al., 2012 [10]	Microarray RT-qPCR (miR-132-3p)	NS	Decreased	NS
	Hu et al., 2019 [11]	Small RNA-seq	NS	NS	NS
STG (BA22)	Beveridge et al., 2008 [12]	Microarray	NS	NS	NE
	Beveridge et al., 2010 [7]	Microarray	NS	NS	NR
Amygdala	Liu et al., 2018 [13]	Small RNA-seq	NS	Decreased	NS

(a/b/c) indicates specific miR-29 variant reported in each study. Abbreviations: BA, Brodmann’s area; DLPFC, dorsolateral prefrontal cortex; NE, not examined; NR, not reported (unclear if NE or NS); NS, not significant; STG, superior temporal gyrus.
